# A test-retest dataset for assessing long-term reliability of brain morphology and resting-state brain activity

**DOI:** 10.1038/sdata.2016.16

**Published:** 2016-03-15

**Authors:** Lijie Huang, Taicheng Huang, Zonglei Zhen, Jia Liu

**Affiliations:** 1 State Key Laboratory of Cognitive Neuroscience and Learning & IDG/McGovern Institute for Brain Research, Beijing Normal University, Beijing 100875, China; 2 Beijing Key Laboratory of Applied Experimental Psychology, School of Psychology, Beijing Normal University, Beijing 100875, China

**Keywords:** Brain imaging, Functional magnetic resonance imaging, Intelligence, Computational neuroscience

## Abstract

We present a test-retest dataset for evaluation of long-term reliability of measures from structural and resting-state functional magnetic resonance imaging (sMRI and rfMRI) scans. The repeated scan dataset was collected from 61 healthy adults in two sessions using highly similar imaging parameters at an interval of 103–189 days. However, as the imaging parameters were not completely identical, the reliability estimated from this dataset shall reflect the lower bounds of the true reliability of sMRI/rfMRI measures. Furthermore, in conjunction with other test-retest datasets, our dataset may help explore the impact of different imaging parameters on reliability of sMRI/rfMRI measures, which is especially critical for assessing datasets collected from multiple centers. In addition, intelligence quotient (IQ) was measured for each participant using Raven’s Advanced Progressive Matrices. The data can thus be used for purposes other than assessing reliability of sMRI/rfMRI alone. For example, data from each single session could be used to associate structural and functional measures of the brain with the IQ metrics to explore brain-IQ association.

## Background & Summary

Magnetic resonance imaging (MRI) is the predominant technique used to study the human brain^1,2^. The same scanner can acquire a variety of image modalities, each of which provides unique information on brain anatomy and function. Among them, structural and resting-state functional MRI (sMRI and rfMRI) have been widely used for characterizing anatomical and functional properties of human brains^3–8^.In particular, measures from sMRI/rfMRI can predict inter-individual variability in behavior and cognition^9–13^. In a large number of these studies, MRI scan is conducted apart from the behavior test, separated by a short- or long-term interval (ranging from several hours to several months^11,14–17^). As brain structure and the temporal dynamics of resting-state activity are likely affected by various factors including social experiences and emotional states^12,18^, an interesting question is whether measures derived from sMRI and rfMRI are developmentally stable over these intervals.

Typically, scans repeated over a long-term interval are not believed to be developmentally equivalent; the developmental effect is considered to be detected with a minimum interval of 3–6 months^19^. Following this convention, in this study, we generated a public dataset to assess the degree of repeatability in anatomical and functional measures from MRI scans acquired over the interval. As part of the Consortium for Reliability and Reproducibility (CoRR) dataset, our data, together with those from 32 other laboratories, has been briefly described in the CoRR overview paper^19^. In this study, we show more details on data collection and quality control. Specifically, the dataset consists of test and retest scans collected with similar imaging parameters from 61 individuals. In each session, sMRI and rfMRI images were acquired from each individual; the interval of repeated sessions ranged from 103 to 189 days. In addition to MRI scans, after the first scan session, each participant took part in a Raven IQ test that measured fluid IQ. Although the similar but not identical imaging parameters is not an ideal setting (i.e., identical scan protocols) to evaluate the long-term reliability of measures from sMRI/rfMRI as non-identical parameters could introduce additional inter-session variance, our test-retest dataset may provide an estimate of the lower bound of the reliability of the measures. Moreover, the data could also be used to assess the relationship between sMRI and rfMRI measures, the relationship between neural measures and inter-individual differences in IQ, and even the relationship between individual inter-session reproducibility of sMRI/rfMRI measures and individual IQ. Finally, in conjunction with other publically available test-retest datasets, our data may help explore effects of imaging parameters on the reliability of sMRI and rfMRI measures. To demonstrate the utility of this dataset, we first calculated a series of quality metrics for both sMRI and rfMRI from each session, such as signal-to-noise ratio and mean frame-wise displacement. We then compared the quality metrics from test and retest data directly.

## Methods

### Participants

Sixty-one students (age: 19.3–23.3, mean age: 21.3; 46 female) from Beijing Normal University participated in the study. All participants were free of psychiatric and neurological problems. Both behavioral and MRI protocols were approved by the Institutional Review Board of Beijing Normal University. Written informed consent was obtained from all participants prior to the experiment. In addition, this study is part of an ongoing project named GEB^2 (Gene Environment Brain & Behavior), which aims to investigate association among neural substrates, cognitive functions, and genetic origins^20–25^.

### Testing procedure

**Figure 1:**
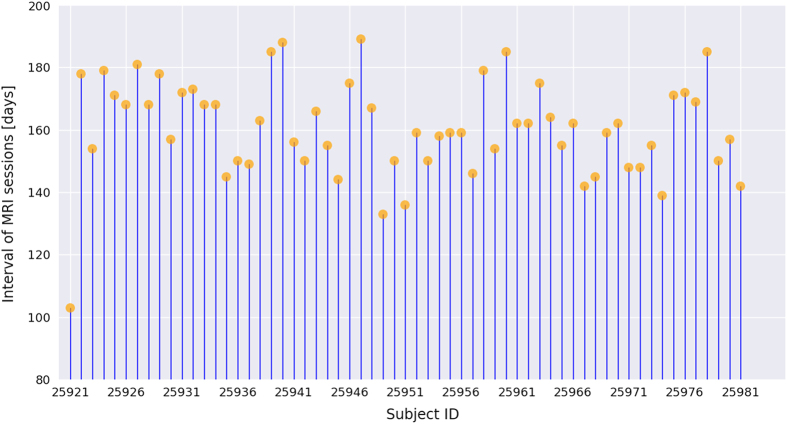
Intervals between two MRI sessions for all participants. Each vertical line corresponds to a participant, and orange disks indicate the interval length.

Each participant was invited to the institute three times. In the first visit, each participant took part in a MRI scan, including a high-resolution sMRI scan and an rfMRI scan. After approximately one month (average =38.4 days, s.d. (SD)=14.8 days), the participants returned to the institute for Raven IQ testing. On the third visit, a retest MRI scan was conducted for each participant with a similar scanning protocol. The imaging parameters of the second scan were slightly different from those of the first scan because the data were not originally designed to evaluate the reliability of MRI measures; instead, the datasets were from two different studies. The interval between the two MRI sessions ranged from 103 to 189 days (average=160.5 days, SD=15.6 days). All participants except one were scanned with an interval more than 130 days, and the intervals of the two MRI sessions were summarized in Fig. 1. Details of each test are described in the following sections.

### MRI scan

All MRI scans were performed using a 3 T whole-body MR scanner (MAGENTOM Trio, a Tim system, Siemens) with a 12-channel phased-array head coil at BNU Imaging Center for Brain Research, Beijing, China. Acquisition parameters of the relevant sequences are summarized below.

#### sMRI scans

In the first session, a T1-weighted magnetization prepared gradient echo sequence (MPRAGE) was used to acquire the high-resolution structural images: field of view (FOV)=256×256×170 mm^3^, imaging matrix=256×256×128, voxel size=1×1×1.33 mm^3^, Time of Repetition (TR)=2530 ms, Time of Echo (TE)=3.39 ms, Time of Inversion (TI)=1100 ms, Flip Angle (FA)=7°, Bandwidth (BW)=190 Hz/Px.

In the second session, the same 3D MPRAGE sequence was used to generate high-resolution structural images, but with slightly different parameters: FOV=256×256×176 mm^3^, imaging matrix=256×256×176, voxel size=1×1×1 mm^3^, TR=2530 ms, TE=3.45 ms, TI=1100 ms, FA=7°, BW=190 Hz/Px.

#### rfMRI scans

In each session, a resting-state fMRI scan was acquired using a T2*-weighted gradient-echo echo-planar-imaging (GRE-EPI) sequence. Participants were instructed to ‘Relax without engaging in any specific task, and remain still with your eyes closed during the scan.’ In the first session, the rfMRI scan lasted 8 min and the imaging parameters were as follows: TR=2000 ms, TE=30 ms, FA=90°, number of slices=33, imaging matrix=64×64, FOV=200×200 mm^2^, voxel size=3.125×3.125×3.6 mm^3^, BW=2520 Hz/Px.

The second rfMRI scans were acquired for each participant with the same instruction and GRE-EPI sequence but with slightly different parameters: TR=1500 ms, TE=30 ms, FA=90°, number of slices=25, imaging matrix=64×64, FOV=200×200 mm^2^, acquisition voxel size=3.125×3.125×4.8 mm^3^, BW=2520 Hz/Px. The scan lasted 10.5 min.

### Raven IQ test

**Figure 2:**
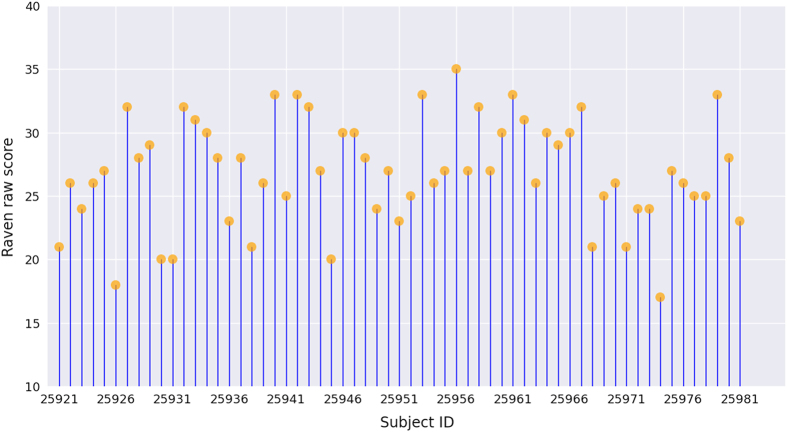
Raven raw scores of all participants. Each vertical line corresponds to a participant, and orange disks indicate the raw scores from the Raven APM test.

Participants’ fluid IQ was measured using Raven’s Advanced Progressive Matrices (APM) test, as participants were highly homogeneous (i.e., college students). The APM test contains 36 multiple-choice items of abstract reasoning, in which participants were asked to identify the missing figure required to complete a larger pattern. Thirty minutes were allotted for the test, and the number of correctly answered items was used as a measure of each individual’s general cognitive ability. The raw scores on the APM test for the 61 participants ranged from 17 to 35, with a mean of 26.9 (SD=4.1). To describe the distribution of the acquired Raven scores, we plotted the raw scores of all participants (Fig. 2). There was a high variance among participants, suggesting that the test was sensitive to individual differences in fluid IQ. Note that only the raw scores from Raven's APM test are presented in the figure. The IQ equivalent scores generated from the raw scores are also provided in the phenotypic data sheet. The corresponding IQ was calculated based on norms for undergraduate students^26^ (mean=22.17, SD=5.60) by z-score processing:

$$z_{i}=\left(x_{i}-M\right)/S$$

$${\mathrm{IQ}}_{i}=100+z_{i}\times 15$$

where M is the mean of the normative sample, S is the SD of the normative sample, x_i_ is the Raven raw score of participant i, z_i_ is participant i’s z-score, and IQ_i_ is the corresponding IQ estimate.

## Data Records

All data records listed in this section are available from the CoRR consortium (DOI: 10.15387/fcp_indi.corr.bnu2, Data Citation 1). In accordance with prior FCP/INDA policies, all NIfTI files were anonymized to remove any information that could identify participants.

### Phenotypic information and Raven IQ scores

Location: BNU_2_phenotypic_data.xls as supplementary table

File format: Excel file.

Basic demographic information including sex, age at the first scan, and handedness is provided in the Excel file. The Raven raw score, IQ score generated from Raven score, and interval between two MRI sessions for each participant are also provided in the file. Moreover, following standard CoRR protocol, some scanning information is included in the data sheet to facilitate data aggregation across sites. The information consists of the category of visual stimulation during rfMRI scans, design of the test-retest scans (i.e., within session or between session), tasks that the participants undertook before the rfMRI scans, duration of first MRI scan and Raven test, and season in which the scans were conducted.

### sMRI scans

Location: sub<ID>/session_[1–2]/anat_1/anat.nii.gz

File format: NIfTI, gzip-compressed.

### rfMRI scans

Location: sub<ID>/session_[1–2]/rest_1/rest.nii.gz

File format: NIfTI, gzip-compressed.

The scan parameters can found in the file BNU2_[test/retest]_scantable.pdf.

## Technical Validation

All 3D volumes were visually inspected at the time of acquisition to check for severe head motion or other potential artifacts. No severe artifacts were observed. To further quantitatively validate the technical quality of the dataset, we calculated a series of quality metrics for both sMRI and rfMRI from each session. These metrics overlap with the ones used by the CoRR overview paper^19^, enabling easier comparison with datasets from other sites in the consortium. The metrics were calculated with the same protocol as the overall CoRR datasets (http://preprocessed-connectomes-project.github.io/quality-assessment-protocol/). We then evaluated the test-retest reliability of the scan quality by assessing the similarity of metrics from two sessions.

### Quality of sMRI

To evaluate the quality of the sMRI scans, we calculated a series of metrics. The definitions and calculations of these metrics are introduced below:

1. Signal-to-noise ratio (SNR). It was defined as the mean within gray matter values divided by the standard deviation of the air values.
2. Smoothness of voxels. It was calculated as the full-width half maximum (FWHM) of the spatial distribution of image intensity values.
3. Contrast-to-noise ratio (CNR). It was calculated as the mean of the gray matter values minus the mean of the white matter values, divided by the standard deviation of the air values.
4. Foreground to Background Energy Ratio (FBER).

**Figure 3:**
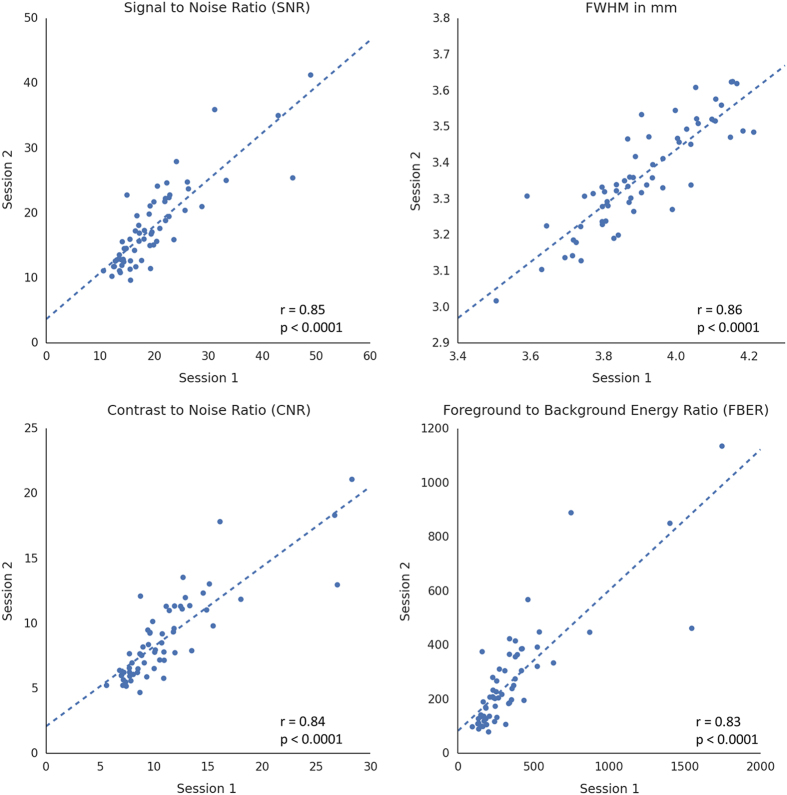
Test-retest plots of quality metrics derived from sMRI images. The blue line indicates the correlation between two sessions across all participants.

For each metric, we first calculated the inter-session correlation across all participants to assess the stability of the sMRI scans. As shown in Fig. 3, all metrics of scan quality show strong correlations between two sessions. The results demonstrated no prominent discrepancy between the test and retest scans as a whole, and that the scans were conducted under a good experimental control.

Other than the inter-individual variability, we further compared the mean of these metrics from two sessions to quantitatively evaluate the difference between two sMRI scans. Table 1 shows that significant differences were found for all metrics derived from sMRI scans, possibly caused by the different imaging parameters. The effect of non-identical imaging parameters on the degree of repeatability of sMRI/rfMRI measures can be further assessed by using the derived reliability from other datasets with identical parameters as baseline.

**Figure 4:**
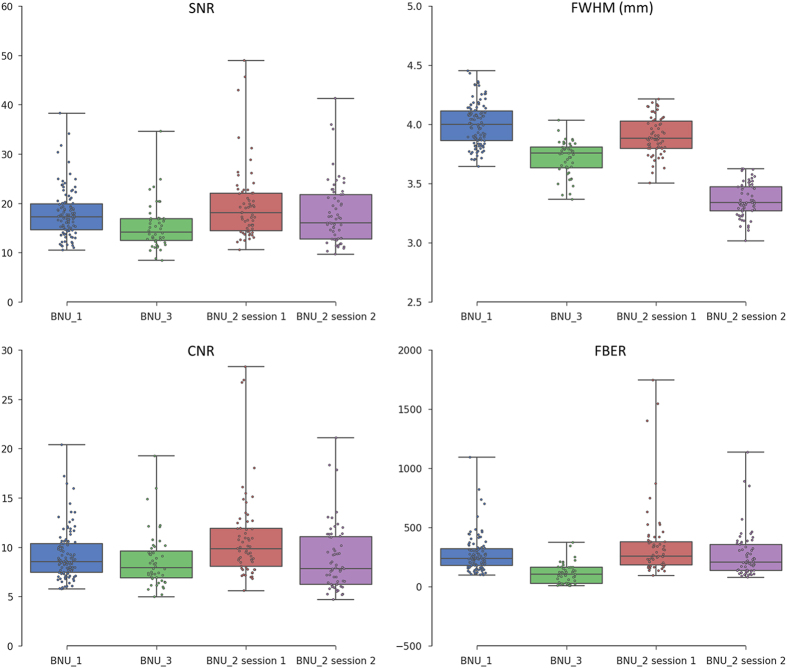
Box plots of quality metrics derived from sMRI images. In order to provide a reference, the metrics derived from datasets BNU_1 and BNU_3 are also shown.

Moreover, in order to provide a reference value for the quality of our data, the metrics of two other public datasets in the CoRR project were calculated (BNU_1: http://fcon_1000.projects.nitrc.org/indi/CoRR/html/bnu_1.html and BNU_3: http://fcon_1000.projects.nitrc.org/indi/CoRR/html/bnu_3.html), which were derived from the same scanner as our data but with identical scanning parameters in both sessions. As shown in Fig. 4, our sMRI data showed similar or even higher quality metrics (i.e., SNR, CNR, and FBER) compared to the other two datasets, suggesting that the quality of our data is acceptable for further processing.

### Quality of rfMRI

A series of metrics were also calculated to characterize the quality of rfMRI scans.

1. Mean frame-wise displacement (FD). A measure of individual head motion that compares the motion between the current and previous volumes. This is calculated by summing the absolute value of displacement changes in the x, y and z directions and the rotational changes about those three axes. The rotational changes are transformed to distance values based on the changes across the surface of a 50-mm radius sphere.
2. Percent of volumes with FD greater than 0.2 mm.
3. Standardized DVARS (D referring to temporal derivative of time series, VARS referring to root-mean-square variance over voxels). The spatial standard deviation of the temporal derivative of the data, normalized by the temporal standard deviation and temporal autocorrelation.

**Figure 5:**
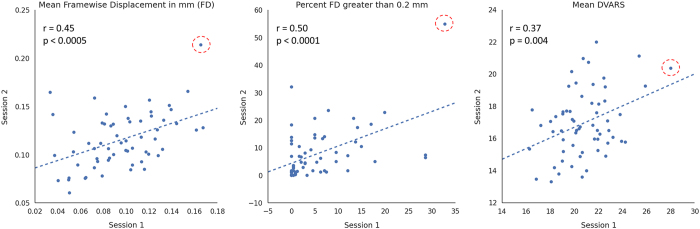
Test-retest plots of quality metrics derived from rfMRI images. Blue line indicates correlation between the two sessions across all participants. Red circle indicates one outlier (ID: 0025932) who had larger head motion during rfMRI scanning.

After calculating the head motion metrics, we first assessed the quality of rfMRI scans with inter-session correlation analysis. We found a moderate test-retest reliability of head motion across participants (Fig. 5). In addition, one outlier (ID: 0025932), who showed greater head motion than others (red circles in Fig. 5), was visually detectable in the plots of MeanFD and Percent FD greater than 0.2mm. The outlier might lead to strong correlation of head motion metrics across participants. Accordingly, we re-computed the inter-session correlation after removing the outlier, and the moderate reliability of head motion was replicated (MeanFD: r=0.38, *P*=0.003; Percent FD greater than 0.2 mm: r=0.31, *P*=0.02; Mean DVARS: r=0.38, *P*=0.003), suggesting that individuals who showed more head motion in test session are likely to have larger head motion in retest session. Similar results were also reported in previous studies^19,27^, and one possibility is that intrinsic psychological factors, such as impulsivity, may contribute to head motion^21^.

Next, we compared the mean of the MeanFD to evaluate the quality of rfMRI scans with two-tailed pair-wise t-tests. As participant 0025932 showed excessive head motion in rfMRI scans, the participant was removed from the analysis. In general, the mean FDs of two sessions were less than 0.2 mm, and only a small percentage of frames showed large FD (about 6.3%). These results demonstrated that the rfMRI data were acquired under a good quality control. Meanwhile, rfMRI images from session 2 showed significant larger mean FDs than that of session 1 (Table 1). The difference may be caused by many possible factors, such as tiredness and restlessness caused by the longer scanning time of session 2. In addition, rfMRI data from session 1 showed a larger DVARS, suggesting that the data had a larger rate of changes in BOLD signals across the entire brain at each frame. The phenomenon may be due to the longer TR (2 s) compared with that of session 2 (1.5 s).

**Figure 6:**
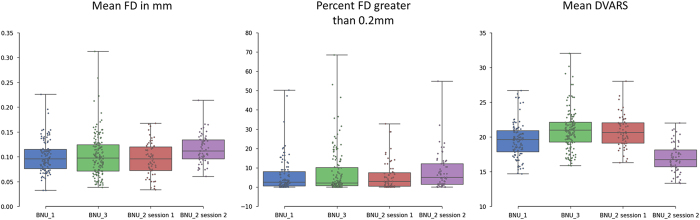
Box plots of quality metrics derived from rfMRI images. In order to provide a reference, the metrics derived from datasets BNU_1 and BNU_3 are also shown.

We also calculated these three quality metrics for the BNU_1 and BNU_3 datasets to provide a reference for assessing the image quality of rfMRI images. As shown in Fig. 6, the rfMRI images in our dataset were acquired with good quality control, showing a similar mean and smaller variance of head motion measures across the participants when compared with the other two datasets.

## Usage Notes

The present dataset can be used to evaluate the test-retest reliability of sMRI and rfMRI measures of other datasets in the repertories of the CoRR. Our data are especially unique because of the long time interval between the test and retest sessions. Thus, the data is particularly suitable for assessment of the long-term test-retest reliability of sMRI and rfMRI measurements. It should be noted that the reliability of the sMRI/rfMRI measures derived from our dataset can only be regarded as the lower bound of the true reliability because non-identical parameters may introduce some additional inter-session variability in the data, and thus cause the underestimate of the true reliability of the derived sMRI/rfMRI measures.

Besides the evaluation of the structural and functional measures from the sMRI/rfMRI scans, the data can serve many other purposes. First, it can be used to assess the relationship between structure and resting-state function at the same time point. Second, the data could aid in assessing the predictability of IQ based on sMRI or/and rfMRI measures. Third, the data can be used to evaluate the reliability in predicting human IQ over different sessions, and even the relationship between the inter-session reproducibility of neural measures and individual IQ. Fourth, when combined with other publically available test-retest datasets with identical parameters, our data can help explore the impact of different imaging parameters on the reliability of sMRI and rfMRI metrics. Finally, another interesting question deals with the reliability of brain region connectivity measures derived from sMRI. Without using diffusion tensor imaging or rfMRI, many studies have shown that inter-regional relationship metrics can be extracted from anatomical images to some extent, which may reflect properties of connectivity information among brain regions^28,29^ and provide new insight to the investigation of brain networks. However, these new metrics highly depends on image quality, and the test-retest reliability is less clear. Our shared data could be used to address this issue.

This dataset is shared in documented standard formats, such as NIfTI or Excel files, to enable further processing in arbitrary analysis environments with no imposed dependencies on proprietary tools.

## Additional Information

**How to cite this article**: Huang, L. *et al.* A test-retest dataset for assessing long-term reliability of brain morphology and resting-state brain activity. *Sci. Data* 3:160016 doi: 10.1038/sdata.2016.16 (2016).

## Supplementary Material

Supplementary Table

## Figures and Tables

**Table 1:** Statistical description of quality metrics (mean±s.e.) from two MRI sessions. Two-tailed paired t-tests were used to compare the means from the two sessions.

**Modality**	**Metric**	**Session 1**	**Session 2**	**Paired** ***t*****-test**
sMRI	SNR	19.88±0.98	17.80±0.82	*P*=0.0001
	FWHM	3.91±0.02	3.36±0.02	*P*<0.0001
	CNR	11.05±0.58	8.81±0.42	*P*<0.0001
	FBER	362.5±40.4	268.6±25.3	*P*=0.0002
rfMRI	Mean FD	0.094±0.004	0.114±0.003	*P*<0.0001
	Percent FD greater than 0.2 mm	5.3±0.88	7.21±0.94	*P*=0.08
	Mean DVARS	20.8±0.29	16.9±0.26	*P*<0.0001
Note: The statistics for rfMRI metrics were calculated after removing the outlier participant 0025932. SNR: signal-noise-ratio; FWHM: full-width half maximum; CNR: Contrast-to-noise ratio; FBER: Foreground to Background Energy Ratio; Mean FD: Mean frame-wise displacement; DVARS: D, temporal derivative of time series, VARS, root-mean-square variance over voxels.				
